# Tackling Root Causes Upstream of Unhealthy Urban Development (TRUUD): Protocol of a five-year prevention research consortium

**DOI:** 10.12688/wellcomeopenres.16382.2

**Published:** 2022-07-08

**Authors:** Daniel Black, Sarah Ayres, Krista Bondy, Rachel Brierley, Rona Campbell, Neil Carhart, John Coggon, Eleanor Eaton, Eleonora Fichera, Andy Gibson, Eli Hatleskog, Matthew Hickman, Ben Hicks, Alistair Hunt, Kathy Pain, Nick Pearce, Paul Pilkington, Ges Rosenberg, Gabriel Scally

**Affiliations:** 1 Population Health Sciences, University of Bristol, Bristol, BS8 1UD, UK; 2 School for Policy Studies, University of Bristol, Bristol, BS8 1TZ, UK; 3 School of Management, University of Bath, Bath, BA2 7AY, UK; 4 Faculty of Engineering, University of Bristol, Bristol, BS8 1TR, UK; 5 Bristol Law School, University of Bristol, Bristol, BS8 1RJ, UK; 6 Department of Economics, University of Bath, Bath, BA2 7AY, UK; 7 Health and Social Sciences, UWE Bristol, Bristol, BS16 1QY, UK; 8 Henley Business School, University of Reading, Reading, RG6 6UD, UK; 9 Institute of Policy Research, University of Bath, Bath, BA2 7AY, UK

**Keywords:** Urban environments, Non-communicable disease, Planetary health, Inequality, Upstream, Commercial determinants of health, Short-termism, Valuation, Power, Decision-making, Risk, Public involvement, Co-production

## Abstract

Poor quality urban environments substantially increase non-communicable disease. Responsibility for associated decision-making is dispersed across multiple agents and systems: fast growing urban authorities are the primary gatekeepers of new development and change in the UK, yet the driving forces are remote private sector interests supported by a political economy focused on short-termism and consumption-based growth. Economic valuation of externalities is widely thought to be fundamental, yet evidence on how to value and integrate it into urban development decision-making is limited, and it forms only a part of the decision-making landscape. Researchers must find new ways of integrating socio-environmental costs at numerous key leverage points across multiple complex systems. This mixed-methods study comprises of six highly integrated work packages. It aims to develop and test a multi-action intervention in two urban areas: one on large-scale mixed-use development, the other on major transport. The core intervention is the co-production with key stakeholders through interviews, workshops, and participatory action research, of three areas of evidence: economic valuations of changed health outcomes; community-led media on health inequalities; and routes to potential impact mapped through co-production with key decision-makers, advisors and the lay public. This will be achieved by: mapping system of actors and processes involved in each case study; developing, testing and refining the combined intervention; evaluating the extent to which policy and practice changes amongst our target users, and the likelihood of impact on non-communicable diseases (NCDs) downstream. The integration of such diverse disciplines and sectors presents multiple practical/operational issues. The programme is testing new approaches to research, notably with regards practitioner-researcher integration and transdisciplinary research co-leadership. Other critical risks relate to urban development timescales, uncertainties in upstream-downstream causality, and the demonstration of impact.

## Introduction and rationale

### Upstream determinants of urban health

**Figure 1.**
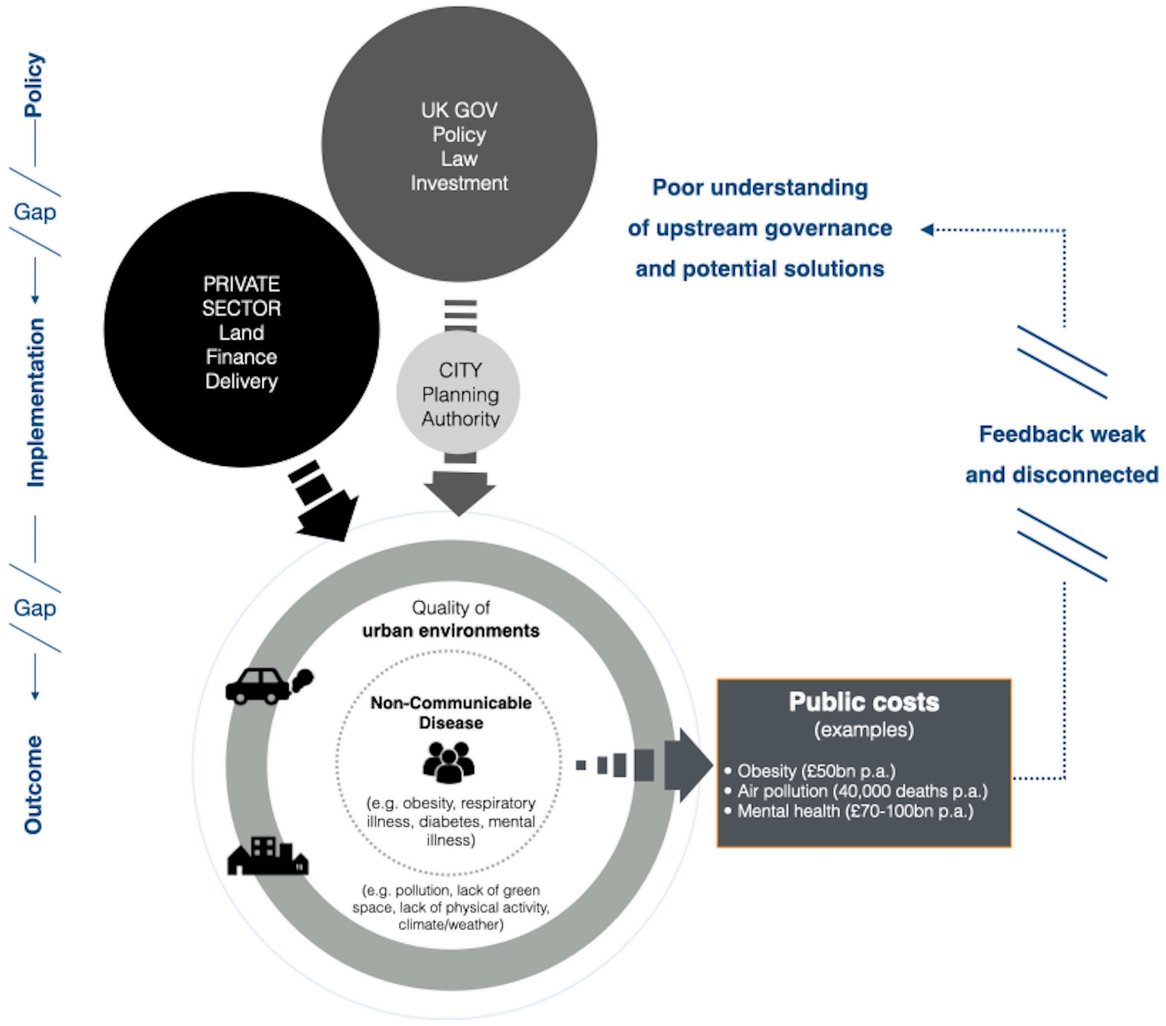
Simplified illustration of Tackling Root Causes Upstream of Unhealthy Urban Development (TRUUD) societal challenge. Public costs from environmental health outcomes downstream are increasingly recognised, but there appears to be a limited understanding of potential solutions.

There is substantial evidence linking non-communicable diseases (NCDs, e.g. respiratory illness, cardiovascular disease, diabetes, mental disorder, cancer) to: the quality of urban environments (e.g. air pollution, noise, lack of green space, physical inactivity, obesogenic food ‘deserts’) ^1– 3^; socio-economic inequalities ^4– 6^; and global environmental degradation, mainly caused by the resource consumption in cities ^7, 8^. NCDs are responsible for 89% of deaths in the UK, most of which are seen as avoidable ^9, 10^. Responsibility is dispersed across many agents ^11, 12^. Fast growing cities are the primary incubators of cultural, social, and political innovation, particularly the UK’s Core Cities ^13^, and local and devolved government plays a pivotal role at the interface between multiple private, public and third sector agencies ^14^. However, the driving force in urban planning and developed in the UK, and across many industrialised nations globally, are large private sector actors - landowners, investors, developers - and political will focused on short, unsustainable timescales ^15, 16^. Increasingly, there is a push towards investigating upstream ^17^, with a particular focus on the ‘commercial determinants of health’ ^6^, the role of the private sector ^18, 19^, and to systems of governance ^20^ - Figure 1. This shift is described as part of a ‘fifth wave of public health’ ^18, 21^ where key problems and solutions are to be found in the domains of, for example, international finance, trade, investment ^22– 27^.

### Economics, valuation, decision-making and risk

Key areas of concern in urban planning are likely to relate to narrow valuation mechanisms, prioritisation, issues of agency and power, short-term horizons, and inequality ^18, 28– 32^. The role of monetary valuation is widely thought to be fundamental; it is a dominant mechanism in decision-making ^22, 33^. However, opinion as to the efficacy of its use in valuing human and planetary health varies ^34– 36^. As the UPSTREAM pilot and other projects suggest, decision-makers are aware that these types of valuations are not comparable to standard cost-benefit analysis, and are used to making decisions with limited information ^30^. Over and above the challenge of effective valuation of externalities, there appears to be little evidence or understanding on how to integrate such external costs specifically into urban planning and development decision-making ^30, 37^. There appears to be a need therefore not only to develop and test new means of valuation targeted at key leverage points ^38– 40^, but also determine the strategic, political, ethical and behaviour shifts needed in corporate governance and associated regulation for prevention to be factored routinely in to core decision-making ^19, 41– 47^.

### Inequality and effective public engagement

Material and power inequalities are primary drivers behind current tensions in society ^4^. Investigating disparities in resource distribution and power dynamics is therefore fundamental ^48, 49^, as is the role of values in governance, and their implications for institutional agents ^19, 44^. Involvement of the lay public in urban planning is already mandatory and has been for many decades, yet societal impact remains limited ^50– 53^. A further challenge therefore relates to how meaningful and effective communication on health inequalities can take place between the lay public downstream and the complex systems of decision-making upstream ^54^. Issues include: the complexity of the problem, the wide range of disciplines involved, the resource needed for effective co-production, the core focus on real-world impact, and the need for effective bridging between academia and practitioner groups. New approaches to research management, clear understanding of context, and the use of the creative arts in surmounting these barriers to communication may be part of the solution ^55, 56^.

### Complexity, causation and the need for new approaches

Addressing these highly complex challenge areas requires new approaches to research ^18, 57^ based on strong theory that not only embrace systems approaches ^58– 60^, inter-/trans-disciplinary working and co-production with real world decision-makers and impacted stakeholders ^61, 62^, but also that have societal impact as a strategic goal and critical reflective practice as a central mechanism ^57, 63^. Systems approaches can enable researchers to navigate complexity, put ‘strategic investment of energy in particular parts and processes of the system’ ^38^, and enable navigation of complexity by making ‘implicit mental models explicit’ ^31^. Yet intervening in urban governance ‘systems of systems’ is not straightforward ^64^. It requires us to ‘balance between complexity and the reduction of that complexity’ and to ‘weigh the costs and the benefits’ ^65^. Research design in this space must look beyond simply the application of systems approaches ^29, 66^. There is a need for: state and non-state actors to work together ^67, 68^; governance of multi-actors settings without reverting to command and control (‘meta-governance’) ^25, 69^; recognition of local governments' limitations ^30^ and the dominant roles of the many and varied private sector controlling agencies and the wider political economic landscape ^6, 19, 41, 46^. Considerable challenges in better decision-making include not only the inevitable lack of evidence, but also demonstrably linking upstream decision-making with downstream health outcomes ^70– 72^. This is particularly challenging given the length of time it can take for urban developments to be built, the need for political will to change, and the significant uncertainty around future impact ^32, 43, 73, 74^. Researchers must work alongside real-world actors if solutions to these complex global challenges are to be found ^75– 77^. This has potentially profound implications for research governance and leadership, linked reflective practice and critical theory ^78, 79^.

## Protocol

### Research setting

This research focuses on two case study areas of major urban development in the UK, Bristol (City) and Greater Manchester (Combined Authority), and their associated national governance systems: i) large-scale mixed-use (housing and commercial) development in Bristol City Council (BCC); and ii) major transport plans and projects in Greater Manchester Combined Authority (GMCA). Within those areas we will focus both on a) the ‘keystone’ actors and processes that control the primary assets and functions of urban planning and development, working with local government partners, while focusing in particular on the dominant private sector actors, as well as b) the communities and ‘lay’ publics affected by these keystone decision-makers. At the national level, we will engage with actors from the legal, regulatory and wider policy systems, including the third sector. We will undertake at least 200 interviews and 20 systems focus groups.

### Study aims

**Figure 2.**
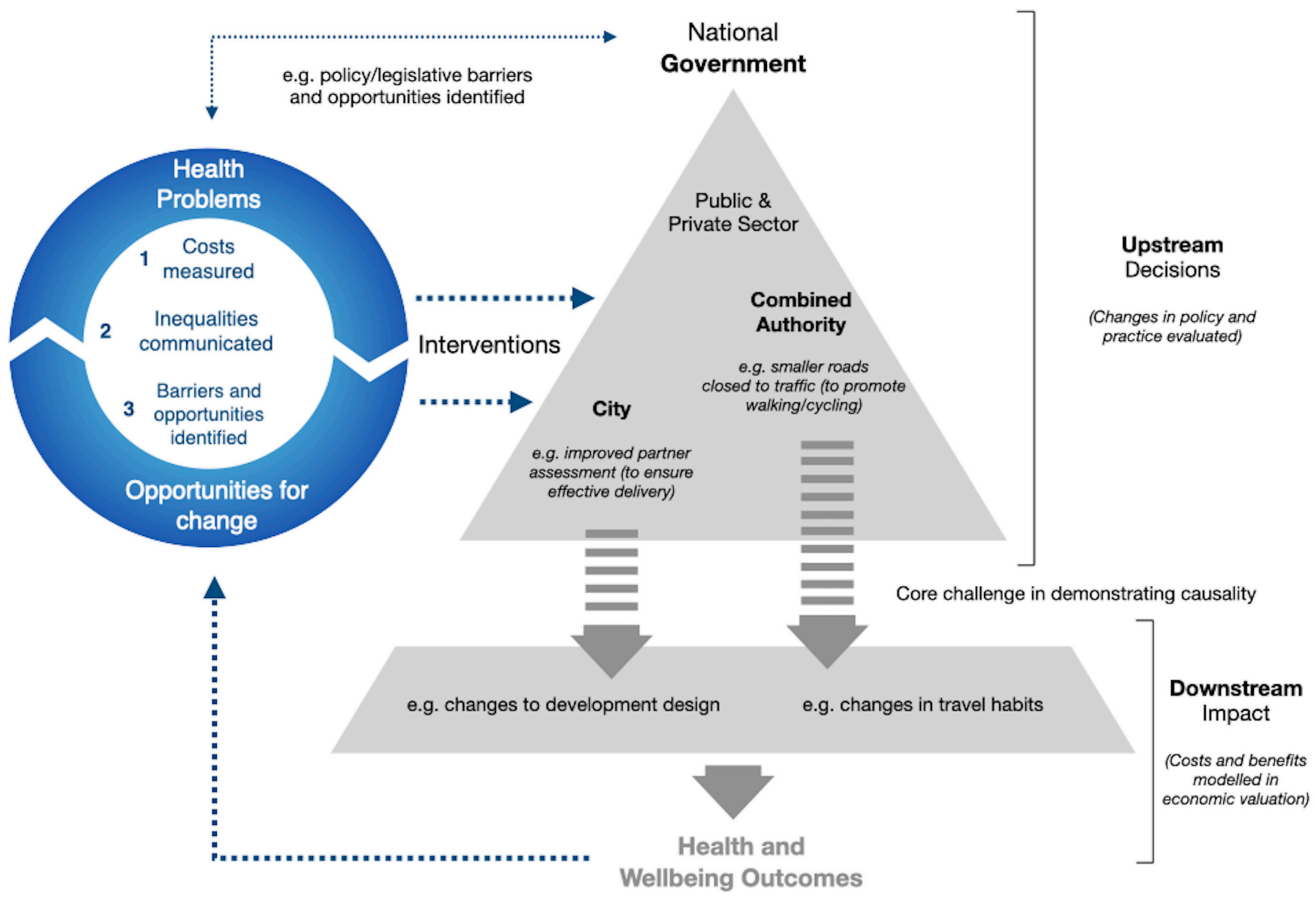
Schematic diagram of the 3-part intervention and potential pathways to impact. Public costs from environmental health outcomes (downstream impact) modelled using economic valuation. Potential solutions – changes in policy and practice (upstream decisions) – mapped and validated with end users. Both combined and communication of health inequalities and presented to decision-makers. Changes in policy and practice evaluated.

Our overarching aim is to develop and test a replicable multi-action and adaptable framework intervention in two large-scale case study urban challenge areas (major transport and mixed-use housing development plans and projects) – Figure 2 - in order to enable a paradigm shift in how health is valued and integrated at root-cause decision-making points.

Our work package (WP) aims are as follows:

- WP1 aims to: map and understand the main drivers of urban development and management; test and refine the co-produced multi-action intervention.
- WP2 aims to discover the type of new evidence that can enhance the economic valuation of health impacts associated with the urban environment.
- WP3 aims to maximise societal impact through strategic coordination of the research programme and co-production of the intervention.
- WP4 aims to develop and test professionally produced, citizen-led creative arts projects that will help decision-makers to understand better the challenges of health inequality.
- WP5 aims to enable a comprehensive programme of knowledge exchange, and monitor and evaluate the impact strategy.
- WP6 aims to maximise the efficacy of inter- and trans-disciplinary working and impact planning.

### Research question, primary objectives and study design

Our overarching research question is: how might prevention of risk factors causing NCDs and negative planetary health outcomes be fully incorporated by those with the most control of urban development in the UK? Our primary objectives are:

1. To engage the wide range of actors involved in shaping decision-making in urban planning and development within our two case studies.
2. To map and understand the systems of urban development decision-making across central and city-regional government, and communicate these to identified stakeholders, including the lay public.
3. To co-produce, and test with a wide range of stakeholders an intervention and evaluation framework, embedded in robust societal impact strategy, made up of three areas of evidence, and targeted at critical points of leverage within these urban development systems:
  i. *Health improvements:* Modelled economic valuation of changed health outcomes, linked to those responsible for payment.
  ii. *Opportunities for change:* mechanisms identified and tested with users and stakeholders for improving policy and practice in both private and public sector, at local and national level.
  iii. *Health inequalities:* Citizen-led, professionally curated creative arts outputs that represent the life experience, views and wishes of those suffering from health inequalities.
4. To deliver a highly impactful knowledge exchange programme with our broad range of users and advisors to ensure long-term health improvement beyond the five year TRUUD programme.

**Figure 3.**
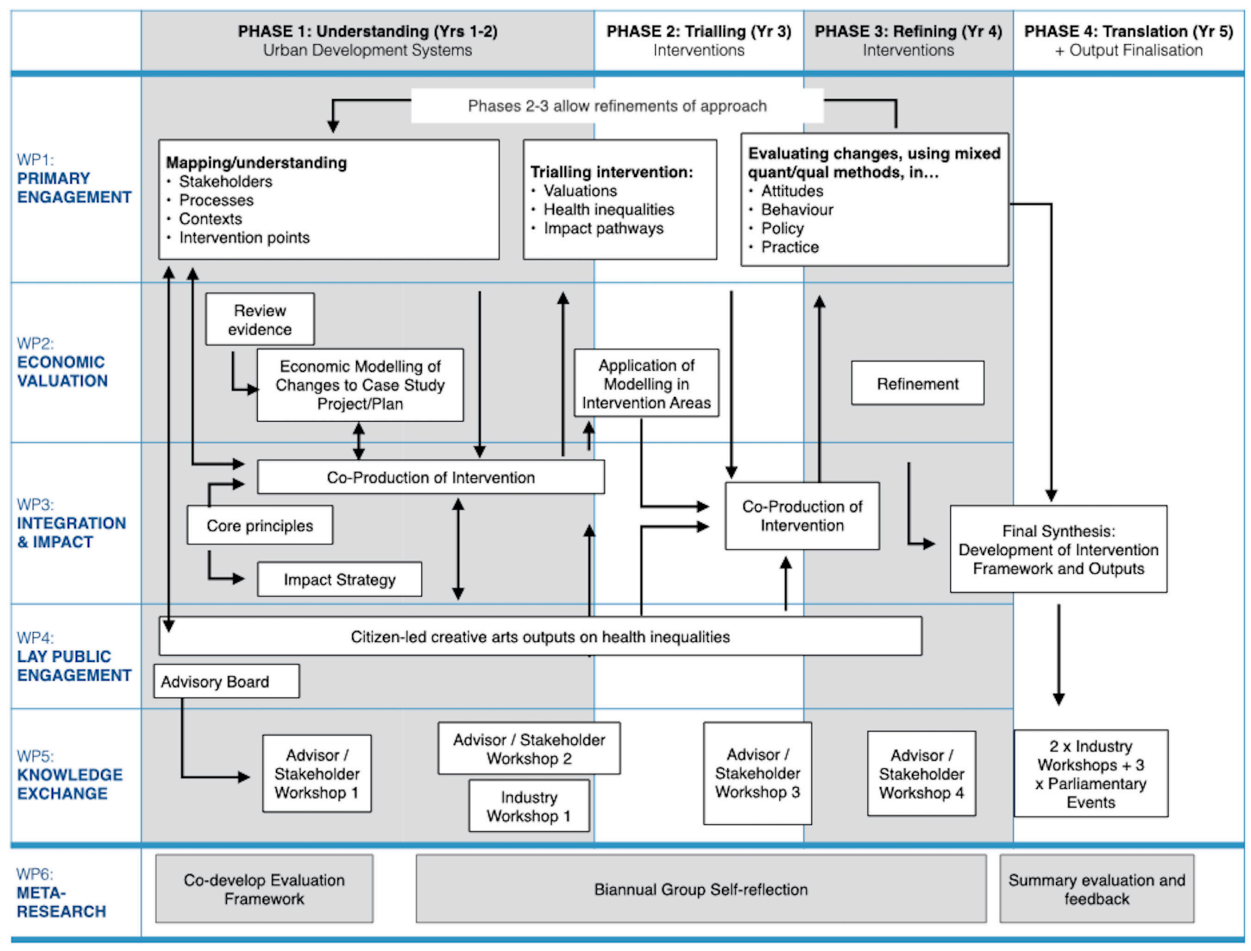
Simplified flow diagram showing work packages (WP), phases and main activities. Main research WPs are WPs 1, 2 and 4. WP3 integrates whole, WP5 externally facing, WP6 to enable group self-reflection and improvement. Programme highly integrated across all WPs – arrow only illustrative of interactions.

The programme comprises six fully integrated and overlapping work packages (WP) delivered concurrently over four sequential programme phases of engagement - Figure 3:

- Phase 1: Mapping and understanding of systems
- Phase 2: Developing and testing of intervention (evidence and approach)
- Phase 3: Refining the intervention
- Phase 4: Translation (final knowledge exchange)

The programme involves substantial co-production and knowledge exchange throughout, via interviews, focus groups, embedded researcher engagement and observations, industry roundtables, annual advisor conferences, parliamentary events and linked ad hoc engagement. End-user testing is central to the design throughout; we will follow an iterative process of co-production, evaluating the changes in attitudes, behaviours, policy and practice amongst our target users, and refining and testing the intervention accordingly.

### Programme coordination and work packages

The six work packages are highly integrated and interdependent. WPs 1–4 make up the core research activity: stakeholder engagement, primary data collection and analysis (WP1); economic valuation (WP2); programme integration, intervention co-production and impact-orientation (WP3); lay public engagement, creative arts and health inequalities (WP4). WP5 is responsible for internal and external communications, knowledge exchange and impact evaluation across the programme. WP6 is tasked with supporting and enabling the consortium to reflect upon and improve their research practice. 

### WP1: Decision-maker engagement and intervention testing

*Methods:* Phase 1 data collection will focus on understanding and mapping the case study systems: actors, mechanisms, data used, power dynamics, dominant drivers/incentives, priorities, wider influencers, boundaries, dependencies/interdependencies, barriers, ’tipping’ points, and regulations. It will also include the extent to which: a) the public are already involved in decision making at a strategic level, and b) decision-makers understand how their decisions impact on local communities. Methods employed will include:

a) Actor mapping – inclusion criteria: perceived level of influence in large-scale property development and transport planning: public (policymakers, politicians, civil servants, council officers); private (asset owners, financiers, agents, consultants); third sector (social housing providers, NGOs); lay public experientially affected by linked aspects of the urban environment (e.g. lack of access to green space, air pollution, noise).
b) Literature review - search terms based on findings from open investigation across multiple disciplines; ten core concepts: ‘power’, ‘governance’, ‘institutions’, ‘change management’, ‘networks’, ‘decision-making’, ‘type/ use of evidence’, ‘systems thinking’, ‘value/valuation’ and ‘risk management’; inclusion criteria in the form of four research questions focused on: robustly defining these ten concepts, key conceptual insights, theories used, and interventions used/suggested. (An initial scoping exercise, the main purpose of this was to enable the newly recruited group to familiarise themselves with each others’ knowledge domains; multiple databases from each of the disciplines are used).
c) Semi-structured interviews (>200) – purposive sample recruited primarily through existing networks; additional participants identified through snowball sampling; individuals approached by telephone and in writing to inform them of the project, invite their participation; interviews to be conducted in short term and where necessary (e.g. COVID-19) via video-conference, face to face where possible; interviews conducted by experts in the field matching those of the participant to ensure strong rapport with participants and richest possible data.
d) Observations – undertaken by two full time participant observers (PO) seconded part time to Bristol City Council (Growth, Investment and Infrastructure) and Greater Manchester Combined Authority (Research Division) respectively; data to be gathered constantly, recorded in research diaries (consistent across both case study sites), on ten core concepts; supported where possible by documentary evidence (e.g. meeting minutes).
e) Systems workshops (>20) coordinated via WP3 – participants identified and recruited as with (and following) interviews; identified knowledge holders to discuss and debate the findings, and help to provide additional clarity on data gathered through the interviews, earlier focus groups, and observations; workshop purpose and focus directed by focus area research lead; task management, workshop design and facilitation support provided by group systems engineers; workshops conducted via video conference; timing will vary depending on purpose, but typically will require three hours; various online software will be used (e.g. Miro, Mural) designed for the purpose of collective ‘whiteboarding’ and generation of systems diagrams.

Regarding private sector engagement, focus on corporate governance will go beyond standard functions - marketing, strategy, and financial reward - to include overarching issues relating to prevention of ill-health: strategic priorities; ownership; distribution of surplus profit; horizon of decision-making; control (instruments and exercise); commitment to ‘Environmental, Social, and Governance’ (ESG); structure and functioning of the Board of Directors ^44, 45^. Industry focus groups will be co-coordinated with membership bodies, NGOs and invited senior practitioners.

*Outputs:* The main outputs from WP1 will be from interview and focus group preparation and outputs, including: literature and policy reviews, graphic visualisations, findings reports and analysis; a public health assessment tool for corporations involved in urban development, which will be of use to public and private sectors to align their own organisational structure to long-term health outcomes, and to improve their procurement and partnership strategies; guidance on organisational models and structures that are better aligned with long-term social and environmental health.

### WP2: Economic Valuation

*Methods:* An extensive critical evaluation of the current literature (focused on quality, uncertainty, validity of method) will enable us to construct a single-source database and modeling tool of the quantitative and economic evidence, building on previous work ^80^. It will incorporate academic and grey literature and will utilize a quality-based classification system and grade the associated uncertainty. Principal search terms include: ‘Valuation of health disutility’, ‘willingness to pay’, ‘cost of illness’, ‘non-market valuation’ and ‘health preferences’. Inclusion criteria include: the alignment of the health outcome definition in the valuation study with that in the epidemiological health impact studies; English language; publication in the previous 20 years, and; being a primary research study. Primary databases to be searched include: Econlit, Scopus, Web of Science, and IBSS. These valuations will use a combination of both market and non-market (revealed and stated preference) methods to estimate the cost components of health impacts. It will enable a more comprehensive coverage of health in economic appraisal than that existing to date which has primarily focussed on the air quality context ^81^. Existing tools used by decision-makers – primarily spreadsheet models - that utilise the data generated in this WP will be used to generate cost-benefit estimates of individual urban development design decisions as well as policy-level decisions. Costs associated with urban form will be derived from the range of stakeholders involved in the project, and will be based on publicly available market data (e.g. pollution; noise; modal share; green space).

*Output:* The main output will be an interactive and adaptable economic database model applicable in a range of core decision areas, which will be a core part of the overarching TRUUD decision support framework.

### WP3: Programme integration, impact-orientation and co-production of interventions

**Figure 4.**
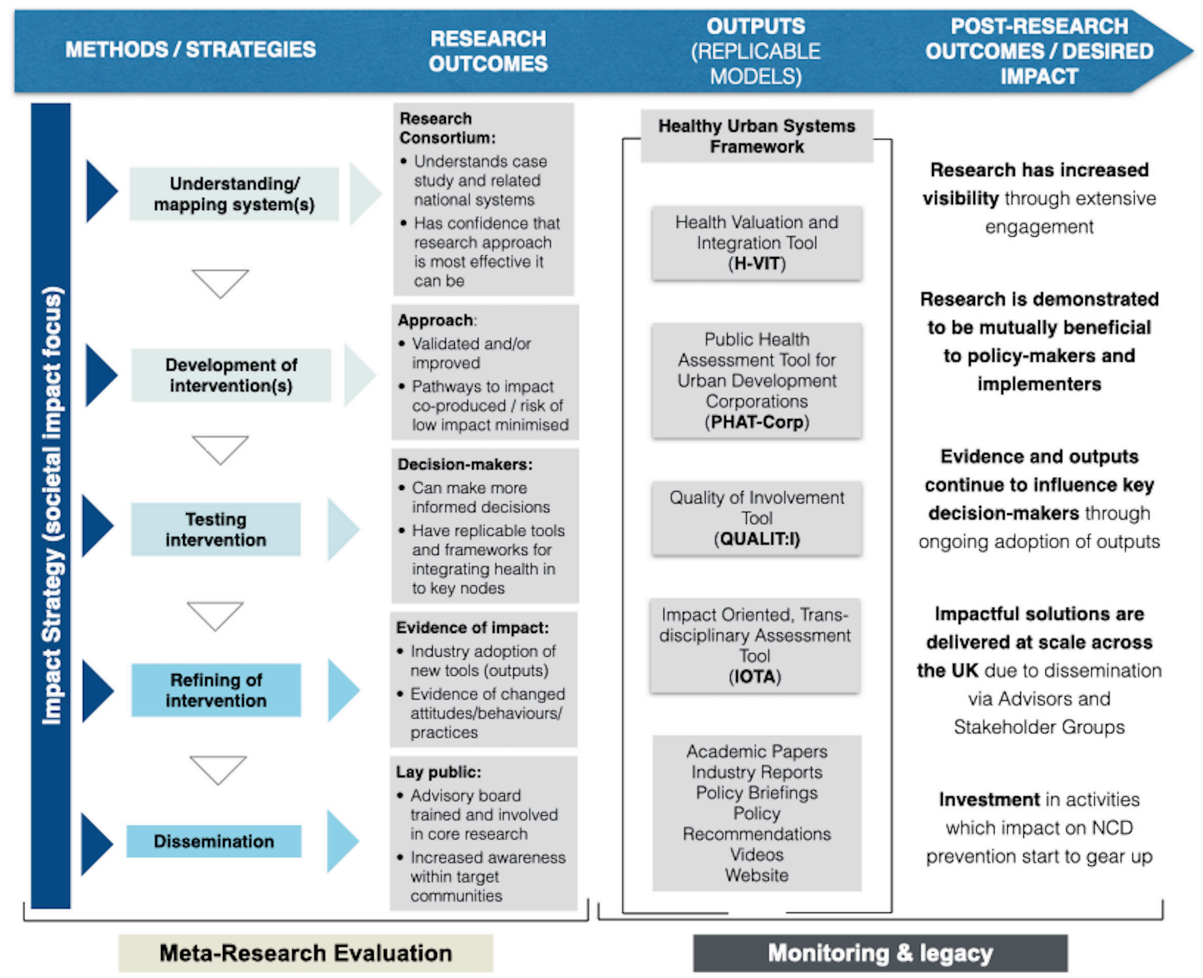
Tackling Root Causes Upstream of Unhealthy Urban Development (TRUUD) theory of change (ToC) modelled on the UK Prevention Research Partnership (UKPRP) ToC. In addition to main outcomes during award period and post-award, the TRUUD ToC addas an explicit impact strategy (focused on societal impact) input across the programme, as well as a meta-research evaluation of methods and award outcomes, and monitoriting and legacy of outputs and post-award outcomes. NCD – non-communicable disease.

*Methods:* Phase 1 focuses on the development of shared understandings across the newly formed consortium ^29, 57, 78, 82^. This includes formalization of: the theoretical foundations underpinning the UK Prevention Research Partnership; how foundational principles – trans-disciplinarity, societal impact, need for new approaches - are embedded in TRUUD’s research governance and operationalization; key definitions and understandings (e.g. ‘events’ in ‘complex social systems’) ^18, 58, 72, 78^. Concurrently, co-production of interventions requires an initial focus on early programme integration and management, through co-development with WP Leads of detailed, interdependent implementation plans for WPs 1, 2, 4 and 5. The detailed WP3 implementation plan will then build on the early theoretical framing and will include strategic considerations in programme integration and detailed steps for co-producing the multi-action intervention at the end of each phase. A core innovation in this WP is the integration of the multiple approaches in to a soft systems framework for evaluating causal links and probabilities between decisions made far upstream and health outcomes downstream based on the hierarchy of research impact ^83– 86^. The intervention, impact and evaluation strategies will be co-produced and tested iteratively internally and externally with end users. In this way we are in accordance with latest MRC/NIHR guidelines on complex interventions, given we will be working with stakeholders to assess which outcomes are the most important and agree how to deal with multiple outcomes in the analysis ^87^ Example evaluation methods we will be employing include: group model building, social network analysis, economic modelling, and qualitative interviewing and workshop-based data gathering. The implementation plan will require sufficient flexibility such that intervention design can respond to a wide range of upstream contexts and applications that will be identified during the first and second rounds of engagement (decision tools span multiple actors and sectors and may include, e.g.: corporate strategy, KPIs, risk management, Cost Benefit Analysis, Multi-Criteria Analysis (MCA), Red Book Valuation or Green Book Valuation) ^88– 90^. Initial development will take place in Phase 1 with refinement in Phases 2–4. The programme as a whole will be underpinned by a strategic impact planning and evaluation framework drawing on Fast Track Impact templates (stakeholder analysis, impact planning, impact monitoring) and Mission-Oriented Research, ensuring integration with our working theory of change and pathways to impact models, and alignment to the UK Prevention Research Partnership (UKPRP)’s broader theory of change – Figure 4. These combined strategies will draw on emerging best practice in the fields of the ‘Science of Team Science’, including the Four-Phase Model of Transdisciplinary Team-based Research ^62, 91, 92^.

*Outputs:* The main outputs from this work package are: i) a book or monograph aimed at the academic community and taking a critical theory lens to the call for new approaches to researching complex societal challenges, considering governance, operationalisation and structural barriers in particular, using TRUUD as a case study; ii) a Health Valuation and Integration Toolkit (H-VIT) with Strategy Guidance Note aimed at city, region and national level decision-makers in public and private sector, which bridges the economic database model (from WP2) to urban governance contexts, and provides a framework for its application with wider, qualitative valuation techniques; iii) a paper (or papers) reporting on the findings from integrating multiple approaches in the development and evaluation of a multi-action intervention across multiple sectors and systems of decision-making.

### WP4: Citizen-led communication of health inequalities through the creative arts

*Methods:* The creative outputs from WP4 will therefore need to meet two design criteria – they need to be: 1) authentic in terms of articulating the impact of health inequalities on the lives of people living in underserved communities; 2) impactful in terms of relevance to upstream decision makers and their core values. In Phase 1 the WP4 team will focus on developing an understanding of: a) decision-makers’ core values and how they think of health inequalities (in collaboration with WP1); b) what creative arts approaches will be most effective in communications. In order to achieve this the WP4 team will co-design the creative outputs with input from both upstream decision makers and community representatives and organisations. Grounding their work in good quality involvement practice (UK Standards for public involvement), the team will work closely with two community-based organizations in Manchester and Bristol, Live Well, Make Art (LWMA) and Knowle West Media Centre (KWMC). The team will: recruit community representatives – initially, two from each location - to help us co-design the creative outputs; develop a brief for the production of the creative outputs, informed by the evidence generated by WP1 and identify and approach upstream decision makers to participate in the co-design of the outputs (based on advice from the External Advisory Board and via WP1 networks). Recruitment will be based on possession of relevant skills and lived experience within the communities we are working with. We will, as far as possible, recruit a diverse group in terms of ethnicity, gender and social class. We will recruit approximately 5–6 public contributors in total from each location, who will then form the TRUUD public advisory group. Applications to carry out the work will be open to organizations with a proven record of accomplishment in this area of work, and will be judged by a management group consisting of the WP4 Lead, SRF, TRUUD Director, two community representatives and representatives of LWMA and KWMC. This group will also oversee the co-production of the outputs and ensure that work meets the specifications laid down in the project brief. The output will be incorporated into the TRUUD intervention, presented to decision-makers as part of the second and/or third phase interviews and focus groups (WPs1 and 3), and evaluated in line with work carried by WP3.

*Outputs:* WP4 is responsible for the production of: three professionally produced, community-led creative arts outputs on community health inequalities aimed at decision-makers; a Quality of Involvement Tool (QUALIT:I) designed to help researchers, users and lay publics understand which key stakeholders are involved and to what extent.

### WP5: Knowledge exchange and impact monitoring

Work Package 5 is split in to two sub-WPs: knowledge exchange (WP5a); impact monitoring (WP5b).

***[WP5a: Knowledge exchange]***

*Methods:* In addition to digital and printed communications (website, social media, video, policy briefings), the knowledge exchange programme will employ a broad range of channels of communication designed to maximise two-way flow of information amongst identified stakeholders and advisors. These will include four annual one-day workshops will be held for critical sense-checking and co-production of next steps, including advisory support and Q&A with 100 advisors and stakeholders. There will also be annual meetings with the External Steering Board, and the Lay Public Advisory Board. At least one parliamentary event will be held, targeted at key actors within government and key members from the advisory groups who have committed to engage at national level.

*Outputs:* The main knowledge exchange outputs from WP5 will be both internal and externally facing and will include: a knowledge exchange, communications and social media strategy; high quality policy briefing notes and industry reports aimed at key decision-making groups; three (public sector, private and lay public) 3–8 minute videos clearly articulating in lay terms the identified findings, opportunities, and new research areas; website (with all documents and media).

***[WP5b: Impact monitoring]***

*Methods:* The impact evaluation framework will be developed from the WP3 impact strategy document and will be likewise co-produced by all consortium members, using the Fast Track Impact templates in the first instance ^83^. The impact strategy will have incorporated data from the WP1 stakeholder analysis and will consider both a) users’ interests, influence, motivations and needs and b) the lay public engagement work (WP4; e.g. strong/weak publics) ^83, 93, 94^. The effectiveness of the impact strategy will be monitored throughout the programme across all WPs, with particular reference to key stakeholder groups. The impact strategy will be designed to interface with the knowledge exchange strategy, particularly during the final evaluation in year 5, to maximise long-term societal impact of TRUUD. 

*Outputs:* Impact planning outputs will include the impact strategy (programme coordination), stakeholder analysis (WP1) and impact planning/monitoring frameworks.

### WP6: Meta-research and group reflective practice

*Methods*: An initial literature review has drawn on the Web of Science and Scopus databases. Initial search terms were for ‘meta-research’, ‘transdisciplinary’, and transdisciplinary - ‘research’, ‘framing’, ‘impact’, ‘innovation’, ‘assessment’ and ‘evaluation’. Given limited papers in this area, searches also included consideration of salient papers references and journals. Data is being collected through annual qualitative interviewing and ongoing researcher observation, reflection and analysis from and about the research team themselves and other actors in the research by means of reflective learning logs, worldview and research paradigm assessment by way of questionnaire, and methodology, method, tool and process appraisal for points of interaction and diversion. The sample will include researchers and stakeholders active on the TRUUD project. Participants will fluctuate over time as the meta-study evolves in response to participation in the larger project. There will be three key types of participant: 1) academic partners (<30); 2) research staff recruited to work on the project (typically research associates) (<50); and 3) non-academic city partners (<30). Participant inclusion criteria: role in the TRUUD project; relevance to current foci of WP6 meta-research; proportionate across both academic, public and private sectors and levels of seniority; participants agreement to take part. Exclusion criterion: those not working on TRUUD (including academics, and stakeholder practitioners from the public, private and third sectors). Evaluation and recommendations will take place through all phases of the project to ensure reflective practice across all WPs. The learning from WP6 will comprise two areas. Firstly an internal focus on coproducing: learning how to enhance the effectiveness of the TRUUD research processes and impacts, and understanding the degree to which inter- and transdisciplinary approaches support successful research. Secondly there will be an external focus: using the opportunity presented by the diversity of researcher and scale of research activity aiming to make a wider contribution to UK research effectiveness. This will include investigating how to balance between fully transdisciplinary research and using evidence from single disciplines in delivering impactful outcomes.

*Outputs:* Learning will be captured in an Impact-Oriented Trans-disciplinary Assessment framework (IOTA) designed for those undertaking meta-research of their own complex co-produced research projects.

## Public involvement

Our public involvement strategy starts with the question: how can the lay public be meaningfully involved in complex systems of urban decision-making? Instead of undertaking yet more (often mandatory) consultation with citizens on development proposals, we will establish (in addition to the lay public aspect of the intervention in WP4) a Lay Public Advisory Group (LPAG). The LPAG will include 6 to 12 members who reflect the diversity of the lay publics, they will meet twice a year in each case study location. The LPAG will also participate in the annual wider Advisory Group (WP5); two representative LPAG members will sit on our External Advisory Board. Lay training modules on the challenge areas (e.g. urban health issues, urban planning, power structures) and language will be developed to support the lay advisors and enable them to advise on key areas including interview questions, analysis of qualitative data, development of bridging mechanisms and policy recommendations. Where needed training and lay public learning modules will be provided in upstream urban governance.

## Analysis plan

Analysis of WP1 data will help to inform the design of the multi-action intervention through WP3, and in WP4. Data collected in Phases 2 and 3 will evaluate the extent to which the intervention impacts on policy and practice, and help to develop the next iteration of the intervention, and will be analysed using actor, game and network ^95^, thematic ^96– 98^, and critical, socio-legal and regulatory analysis ^99^, as well as drawing on approaches from systems science, risk management and scenario modelling ^100– 102^. At each tier of governance we will need to identify what is within the control of decision-makers involved in the study (‘endogenous’) and those factors that are largely exogenous (e.g. technological futures, European/global geo-politics) ^40^. Under WP3, soft systems methods will be applied as appropriate (in this and other WPs), and graphic mapping will be employed to enable complex system navigation, understanding and communication. These methods may draw on a range of systems tools and processes including: problem structuring, rich pictures, actor constellations, system dynamics and causal loop models ^103– 106^. The stated preference, survey-based, research in WP2 will draw upon a range of econometric methods to interrogate data that looks to derive monetary values and the determinants of these values. Parameterization of the attendant uncertainties will inform the ways in which data on monetary valuation is subsequently communicated in the case study interventions. In WP6 data will be analyzed iteratively following a Grounded Theory approach ^107, 108^. This will be used to develop a map of concepts and hypothesis relating to the research foci of interest for each phase of the TRUUD work programme. This will form the first stage in the inductive and abductive reasoning to link the TRUUD research findings and impact (outcomes) to the TRUUD research assets, people (academic and stakeholder networks), processes (methods and methodologies) and practices (interventions). If required, statistical analysis will be small-scale and present straightforward statistical analyses on specific issues, e.g. the results of polling and surveys.

## Ethics and risks

Research ethics has been approved by the Research Ethics Committees at University of Bristol’s Faculty of Health Sciences (REFs: 94162 and 10818) and Faculty of Engineering (Ref: 99963), University of Bath’s School of Management (Ref: S21-065) and Economics (S21–034). Where other institutions are involved, they have reviewed relevant ethics applications and decisions and provided written approval. All participants in the research will be asked to provide written informed consent before participation in the research. No individual participants will be identifiable from publications resulting from this study. All data provided will be kept confidential and anonymised. Risks include: change in political administration in partner local governments; alignment of research with real world timescales; effective integration of wide ranging disciplines within limited time and resource constraints; academic structures limiting societal impact. We are mitigating these risks through: embedding researchers in residence; high level of co-production with practitioners; highly integrated research design; governance structure with central coordination function responsible for societal impact strategy. Other risks include: loss of key personnel and case study projects being delayed, which we will mitigate through identification of proxies and identifying a range of potential projects, respectively.

## Dissemination of findings

The research will be disseminated through academic channels (peer-reviewed publications and conference presentations), but also, to maximise the impact of the research, there will be a strong focus on sharing the results of the research with urban development decision-makers. A primary activity in TRUUD is the co-production, with potential end users and stakeholders (including lay public), of the intervention. Throughout there will be an ongoing programme of knowledge exchange through WP5, the strategy for which will also be coproduced with key stakeholders, decision-makers and lay public. We will use a variety of communication channels to reach a broad range of audiences in order to maximise both the societal and academic impact of the research.

## Study status

The research programme started on 1 October 2019. 

## Discussion and conclusion

Identified challenges relate primarily to: a) the effective integration of (the wide range of) researchers and disciplines, and their collective orientation towards a shared societal goal; b) addressing uncertainties in the evidence base; and c) the design and evaluation of beneficial impact on health outcomes (downstream, resulting from decisions made far upstream and in complex real-world situations). This is a large programme of research that brings together many diverse disciplines alongside multiple stakeholders and sectors. We anticipate practical/operational issues, common to all large, highly interdisciplinary projects. The level of complexity and the core focus on demonstrable real-world impact require new ways of working and justifies an additional level of coordination and structuring ^18, 57^. As Hall *et al.* (2014) observe, much of the process of creative endeavour and innovation lies at that point of tension and resolution: *“conflicts and related debate can lead to new perspectives and new knowledge, they ultimately may be helpful for making strategic decisions and enhancing team performance”* ^62^. Conversely, they also warn that *“these differences can result in conflict and negatively impact team performance, if the conflict is not managed”.* In addition to the meta-research work package, we have pervasive reflexive interests stimulated by a shifting emphasis from “public health research” to the broader disciplinary and practical embrace of “health of the public research” ^18^, and will draw on new approaches such as those being pioneered in the field of team science ^62, 109, 110^. The challenge relating to uncertainties links not just to the economic valuation, which is based on assumptions and partial data availability, but also to wider decision factors: full information is rarely available to decision makers, future scenarios maybe unknowable, and therefore decision-making with uncertainty is inevitable. With regards the former, we will ensure that the evidence available for the economic valuation is sourced as comprehensively as possible, but are aware too that significant uncertainties will remain. The challenge for the group will be in identifying salient narratives and developing a supporting framework for decision-making that takes these uncertainties in to account. Risk management approaches are likely to play a prominent part. Within a five-year research project, it will not be possible to demonstrate reduction in NCDs. Apart from abrupt shifts in urban management policies (e.g. congestion charging), urban infrastructure changes slowly, over decades and centuries. The complex range of variables mean that demonstrating clear causation is also impossible. Our goal therefore, is to test demonstrable and immediate changes in attitudes and behaviours amongst the target user groups, as well as changes to policy and practice, within the award period; in particular in decision-making within the systems of governance in Bristol, Manchester (and London in relation to national-level mechanisms linked to city development).

## Data availability

### Underlying data

No data are associated with this article.
