# Supplementary material for: Tackling Root Causes Upstream of Unhealthy Urban Development (TRUUD): Protocol of a five-year prevention research consortium
Source: Wellcome Open Res. 2022 Jul 8;6:30. Originally published 2021 Feb 10. [Version 2] doi: 10.12688/wellcomeopenres.16382.2 (PMC9296993; doi:10.12688/wellcomeopenres.16382.2)
Supplement: Peer review history

# Peer review: Reviewer response for version 2

**DOI:** 10.21956/wellcomeopenres.19993.r51443

**Published:** 2022-07-25

**Authors:** Keisuke Kuwahara

**Affiliations:** 1 Teikyo University Graduate School of Public Health, Tokyo, Japan

At the initial round of peer-review, I provided several comments especially on methodology for clarification. In this revision, the authors have adequately responded to my previous comments and updated their manuscript. Therefore, I have no further comments on the manuscript.

Is the study design appropriate for the research question?

Yes

Is the rationale for, and objectives of, the study clearly described?

Yes

Are sufficient details of the methods provided to allow replication by others?

Partly

Are the datasets clearly presented in a useable and accessible format?

Not applicable

Reviewer Expertise:

NA

I confirm that I have read this submission and believe that I have an appropriate level of expertise to confirm that it is of an acceptable scientific standard.

# Peer review: Reviewer response for version 2

**DOI:** 10.21956/wellcomeopenres.19993.r51444

**Published:** 2022-07-22

**Authors:** Sina Azadnajafabad, Andisheh Amouzadeh

**Affiliations:** 1 Non-Communicable Diseases Research Center, Endocrinology and Metabolism Population Sciences Institute, Tehran University of Medical Sciences, Tehran, Iran; 2 Urban Planning and Design Department, University of Tehran, Tehran, Iran

Although the changes and responses were not utterly satisfying regarding our comments, we suppose the overall efforts of the authors were acceptable at this stage of their proposed protocol of the mentioned project, and this draft earns the merit for indexing in its current format.

Is the study design appropriate for the research question?

Yes

Is the rationale for, and objectives of, the study clearly described?

Partly

Are sufficient details of the methods provided to allow replication by others?

Partly

Are the datasets clearly presented in a useable and accessible format?

Not applicable

Reviewer Expertise:

Public health, Non-communicable diseases, Epidemiology, Health policy, Urban design, Urban planning

We confirm that we have read this submission and believe that we have an appropriate level of expertise to confirm that it is of an acceptable scientific standard.

# Peer review: Reviewer response for version 2

**DOI:** 10.21956/wellcomeopenres.19993.r51442

**Published:** 2022-07-19

**Authors:** Martin McKee

**Affiliations:** 1 London School of Hygiene and Tropical Medicine, London, UK

In my initial comments, I identified a number of issues but recognised that it would be difficult to address them all within what was an already dense paper. I am content that the authors have addressed them, but mainly in their replies to my paper. Given that the purpose of such comments is to inform the research and my review and the replies are on the website, I believe that this is satisfactory.

Is the study design appropriate for the research question?

Yes

Is the rationale for, and objectives of, the study clearly described?

Yes

Are sufficient details of the methods provided to allow replication by others?

Partly

Are the datasets clearly presented in a useable and accessible format?

Not applicable

Reviewer Expertise:

Public Health.

I confirm that I have read this submission and believe that I have an appropriate level of expertise to confirm that it is of an acceptable scientific standard.

# Peer review: Reviewer response for version 1

**DOI:** 10.21956/wellcomeopenres.18021.r46171

**Published:** 2022-01-04

**Authors:** Keisuke Kuwahara

**Affiliations:** 1 Teikyo University Graduate School of Public Health, Tokyo, Japan

This is an elaborate and interesting project involving multiple stakeholders, especially those who are responsible for urban planning and development, exchanging knowledge, and creating arts outputs, in my understanding in order to change the practice on urban planning and development. Even though this is a study protocol paper, details of the protocol are not provided (just showing the overall framework). Thus, it was difficult for me to provide comments. I have some additional comments for clarification as shown below:  

**Protocol:**

- Please clarify whether the study protocol approved by the University of Bristol’s Faculty of Health Sciences Research Ethics Committee is the same as this paper or not. Did the authors submit to the ethics committee a more detailed protocol? Or, did the authors submit the same, one protocol?

**Research setting:**

- Please specify the location of the research setting in this section. Bristol and Manchester?

 

**Programme coordination and workplace packages:**

- Regarding the literature review, MEDLINE (PubMed) was not included. As related papers might be found, it may be better to use MEDLINE/PubMed.
- The authors show a number of interviews workshops. Adding the unit of the number may help improve readability.
- Will authors examine the quality of eligible papers? If so, how?
- I could not find an explanation about methods for evaluating changes in attitudes, behaviors, policy, and practice (WP1-Yr4 in Figure 3). Please clarify.
- Abbreviation of Lay Public Advisory Board is shown as LPAG. Is this correct?

 

**Figure 2:**

- It would be nice if which part indicates “3-part intervention” as there are many arrows.

**Figure 3:**

- WP1 includes evaluating changes in attitudes, behavior, policy, and practice. I want to know their indicators and study design for assessment (e.g., qualitative study, etc.) in this figure.
- To me, it is difficult to understand what “valuations” mean in WP2 from this figure.
- Including words, Phase 1, Phase 2, Phase 3, and Phase 4 in this figure may help improve the connection between this figure and text.

**Figure 4:**

- It would be helpful if the meaning of “AWARD” was clarified.
- I am confused about the timing of yielding OUTPUTS.
- It takes some time to understand the relationship between Figure 3 and Figure 4.

**Other minor comments:**

- 16.  What kind of reporting guideline will be used to report case studies?
- 17.  The authors emphasize “two case studies”. When reporting in academic journals, will the authors merge them or separately report?

Is the study design appropriate for the research question?

Yes

Is the rationale for, and objectives of, the study clearly described?

Yes

Are sufficient details of the methods provided to allow replication by others?

Partly

Are the datasets clearly presented in a useable and accessible format?

Not applicable

Reviewer Expertise:

Public health, epidemiology

I confirm that I have read this submission and believe that I have an appropriate level of expertise to confirm that it is of an acceptable scientific standard, however I have significant reservations, as outlined above.

# Response

**Published:** 2022-07-04

**Authors:** Daniel Black

**Affiliations:** University of Bristol, UK

**Reviewer 3 comment:** This is an elaborate and interesting project involving multiple stakeholders, especially those who are responsible for urban planning and development, exchanging knowledge, and creating arts outputs, in my understanding in order to change the practice on urban planning and development. Even though this is a study protocol paper, details of the protocol are not provided (just showing the overall framework). Thus, it was difficult for me to provide comments.

**Authors’ response:** We are grateful to the reviewers for taking the time to feedback on our Protocol, and apologies for delay in the response. The reviewers’ concerns are understandable and expected, given the wide range of interconnected knowledge domains involved. One of the challenges inherent in developing new approaches to public health research in areas that span multiple knowledge domains outside public health is the challenge of reviewing articles that seek to bring together so many different areas of specialism ( Bammer G, 2013).

 I have some additional comments for clarification as shown below: 

**Protocol:**

 Please clarify whether the study protocol approved by the University of Bristol’s Faculty of Health Sciences Research Ethics Committee is the same as this paper or not. Did the authors submit to the ethics committee a more detailed protocol? Or, did the authors submit the same, one protocol?

**Authors’ response:** The University has its own ethics application form so we did not submit this Protocol itself, but the form sets out, largely, the same details that are in this Protocol. It’s less detailed in some areas (e.g. work coordination), but more in others (e.g. risks/sensitivities). There were also multiple ethics applications submitted later across different faculties and institutions, each having different requirements. University of Bath required a separate approval for the interviews, whereas Universities of Manchester and Reading were happy to approve Bristol’s ethics application. There were also separate applications for the workshops (University of Bristol’s Faculty of Engineering), the economics (University of Bath’s Department of Economics) and a separate survey work for the Frome Gateway (University of Bristol). These new references are now supplied in the revised Protocol.

**Research setting:**

 Please specify the location of the research setting in this section. Bristol and Manchester?

**Authors’ response:** Bristol and Manchester are our two case study city-regions, but we are also engaging nationally with Government and with a wide range of private and third sector actors. We will amend the description to make this clearer.

**Programme coordination and workplace packages:**

 Regarding the literature review, MEDLINE (PubMed) was not included. As related papers might be found, it may be better to use MEDLINE/PubMed.

**Authors’ response:** This was not a formal systematic review, but an initial scoping exercise, the main purpose of which was to enable the newly recruited group to familiarise themselves with each others’ knowledge domains. We used multiple databases from each of the disciplines. MEDLINE/PubMed was one, but there were many others, and most from outside public health. We hope we have made this clearer in the review.

 The authors show a number of interviews workshops. Adding the unit of the number may help improve readability.

**Authors’ response:** We can now add the exact number of phase 1 interviews (127) and workshops (4) now that we have completed them, but we can’t yet specify the number of engagements will be undertaking in Phase 2 so suggest we keep it as originally stated in the Protocol.

 Will authors examine the quality of eligible papers? If so, how?

**Authors’ response:** We did not undertake a formal assessment in the WP1 literature review (such as PRISMA, which we do use in WP2’s systematic reviews) as it was not a formal systematic review. See above comment in response to MEDLINE/PubMed suggestion.

 I could not find an explanation about methods for evaluating changes in attitudes, behaviors, policy, and practice (WP1-Yr4 in Figure 3). Please clarify.

**Authors’ response:** In the methods section of WP3 we state: “ *The intervention, impact and evaluation strategies will be co-produced and tested iteratively internally and externally with end users*.” This echoes MRC’s latest guidelines on complex interventions (Skivington et al, 2021), which states: “ *A crucial aspect of evaluation design is the choice of outcome measures or evidence of change. Evaluators should work with stakeholders to assess which outcomes are most important, and how to deal with multiple outcomes in the analysis*.” They cite as an example group model building and social network analysis, which we are using, but we are also employing health and environmental economic modelling and qualitative interview and workshop data gathering. We have already started evaluating the process through critical reflection and will continue to do so formally through the intervention design and evaluation stages, in order to inform development of programme theory. We are also undertaking ‘evaluability assessments’ as part of the feasibility work. To respond to this concern, we have added some further descriptive text, but it remains the case that it is yet to be co-designed.

 Abbreviation of Lay Public Advisory Board is shown as LPAG. Is this correct?

**Authors’ response:** Thank you for flagging this. It’s not consistent and we have changed. We sometimes use the word ‘Board’ and ‘Group’ interchangeably.

 Figure 2:

 It would be nice if which part indicates “3-part intervention” as there are many arrows.

**Authors’ response:** The whole diagram illustrates the intervention given co-production and testing of the intervention with stakeholders is core to the process, but the circle on the left indicates the three main areas of evidence. We have therefore updated the image to indicate that more clearly and hope this addresses the reviewer’s concern.  

**Figure 3:**

 WP1 includes evaluating changes in attitudes, behavior, policy, and practice. I want to know their indicators and study design for assessment (e.g., qualitative study, etc.) in this figure.

**Authors’ response:** We have yet to detail the evaluation design in detail, which could not be predetermined until it was clear where we would be intervening, not least as a core part of the evaluation needs to be developed with stakeholders. We will likely use a combination of qualitative data gathering through stakeholder engagement (interviews, surveys) alongside the quantitative valuation from the economic modelling, combined with systems approaches (e.g. ripple effect mapping). We have added a few words to that effect in the box indicated, though given there is limited space, it does not describe in full.

 To me, it is difficult to understand what “valuations” mean in WP2 from this figure.

**Authors’ response:** This refers to the health and economic modelling of the two case study plans/projects in Bristol and Greater Manchester, and the development of their real world application in upstream decision-making areas of intervention. We have added this description in to the figure.

 Including words, Phase 1, Phase 2, Phase 3, and Phase 4 in this figure may help improve the connection between this figure and text.

**Authors’ response:** Thank you – that is helpful and we have now updated. We hope this addresses your concern below about the difference between Figures 3 and 4.

**Figure 4:**

 It would be helpful if the meaning of “AWARD” was clarified.

**Authors’ response:** This is the language used by the funder and it refers to the grant/funding period (i.e. the 5 years from October 2019). We have changed ‘award’ to ‘research’.

 I am confused about the timing of yielding OUTPUTS.

**Authors’ response:** This has now been updated to show the Phases and we agree is now clearer, thank you.

 It takes some time to understand the relationship between Figure 3 and Figure 4.

**Authors’ response:** We hope this addresses this concern above.

 Other minor comments:

 16.  What kind of reporting guideline will be used to report case studies?

**Authors’ response:** Given the transdisciplinary nature of our research, we have a range of different disciplines, standards and expectations to accommodate. We will use a range of reporting guidelines depending on the research lead, the target audience and the purpose of the reporting. Typically, our public health researchers would use COREQ for our qualitative research, and we will refer to (and promote) the Equator Network to determine which guideline is most suitable. Our economists may use the CHEERS reporting guideline, though it depends on whether it’s a useful fit as the modelling is a integration of approaches.

 17.  The authors emphasize “two case studies”. When reporting in academic journals, will the authors merge them or separately report?

**Authors’ response:** Both. Some of the researchers will be reporting on just one (for example, the Researchers-in-Residence at Bristol and Greater Manchester), while others will merge them to consider common themes and wider systems implications.

 _______

  

- Skivington et al (2021) A new framework for developing and evaluating complex interventions: update of Medical Research Council guidance. Research Methods and Reporting. *BMJ*. Accessed 22/6/22. Available from: https://www.bmj.com/content/bmj/374/bmj.n2061.full.pdf

# Peer review: Reviewer response for version 1

**DOI:** 10.21956/wellcomeopenres.18021.r46172

**Published:** 2021-10-18

**Authors:** Sina Azadnajafabad, Andisheh Amouzadeh

**Affiliations:** 1 Non-Communicable Diseases Research Center, Endocrinology and Metabolism Population Sciences Institute, Tehran University of Medical Sciences, Tehran, Iran; 2 Urban Planning and Design Department, University of Tehran, Tehran, Iran

We are honored to provide an integrated review of this study protocol based on both public health and urban planning points of view. The authors of this protocol introduced their proposed prevention program to address the unhealthy urban development issue as a root cause of non-communicable diseases (NCDs). In brief, the authors tried to fill the gap in knowledge and executive steps of inappropriate urban development with this research protocol. Although the project and the provided protocol are well thought and designed, we found many major concerns regarding the proposed plan, which comments are as follows in this letter. Additionally, we read the previous comments of the reviewer and the responses to those comments and tried not to emphasize duplicate issues; however, in some cases, it is inevitable as the issue is major and could not be neglected.

 Overall, the draft is too complicated. We understand the nature of such work necessitates providing this much information. However, scientific writing always comes alongside simplicity besides the right and essential components of science, and in case of complexity, even some simple points could be missed and make the research less advantageous.

 Regarding the sections of protocol papers, the format varies based on submitted journals. However, following rational formatting is necessary anyway. Section and subsections of this paper could be divided into three major sections of “Introduction and rationale”, “Protocol”, and “Discussion and conclusion” for a better understanding. It seems that the authors could not follow the coherent presentation of data in the protocol section and the subsections are out of order and this is where the audience of the paper gets bored and confused. Absolutely a revision of the core of this manuscript which is the “Protocol” section could help the reader of this paper.

 “Introduction and rationale”: this section lacks the necessary information about the burden of NCDs in the real world, especially the investigated country, the UK. In fact, similarly, all parts of the paper lack the proper and essential views of public health experts and this is a major shortcoming of the current draft. The provided material is mainly for urban planning experts and policymakers; however, the goal of this project is to reach optimum health among the population. Therefore, adding some details and the language of public health to this manuscript is necessary in this regard, mainly in the introduction and discussion sections, where the importance of this topic and the ultimate results of this project should be highlighted.

 “Protocol”: as the major section of this protocol, this section may cause significant confusion in readers. The information presented in each part of the work packages (WPs) is too much and does not follow a coherent format. A revision of this section to make it more understandable and only bringing practical points in this protocol paper could help more.

 One major concern about this protocol is about the period of this project. Based on available information about the current project, it is planned for five years of action starting in 2019, and at this time, about two years have passed. Publication of a protocol of such a huge project, while it reached about half the way, is an alarming concern. In fact, protocols are meant to provide a clear vision of a prospective project, and publishing a complicated protocol like this in the current approved and executed format could be less useful for the scientific communities of both public health and urban planning. However, some amendments in this manuscript by providing the report and results of what the investigators did in these two years and the achievements they accomplished may be helpful to enable the audience to judge the feasibility and efficacy of such a program. For example, the state and results of the designed workshops mentioned in the protocol should be provided in this manuscript. Since a major time has passed, a progress report is necessary.

 Similar to the previous review, there is still concern about the two cities chosen for the implementation of policies proposed in this protocol. Authors need to clearly state why they chose the mentioned cities for the specific policies. What made the cities special for running the proposed ideas in this project should be elaborated for readers. This point is very important regarding making the study reproducible in other regions and countries of the world.

 “Discussion and conclusion”: as mentioned before, an essential part is missing in this section talking about the potential public health effects of this protocol. A logical estimation of the results and impacts of the implemented policies.

Is the study design appropriate for the research question?

Yes

Is the rationale for, and objectives of, the study clearly described?

Partly

Are sufficient details of the methods provided to allow replication by others?

Partly

Are the datasets clearly presented in a useable and accessible format?

Not applicable

Reviewer Expertise:

Public health, Non-communicable diseases, Epidemiology, Health policy, Urban design, Urban planning

We confirm that we have read this submission and believe that we have an appropriate level of expertise to confirm that it is of an acceptable scientific standard, however we have significant reservations, as outlined above.

# Response

**Published:** 2022-07-04

**Authors:** Daniel Black

**Affiliations:** University of Bristol, UK

**Reviewer 2 comment:** We are honored to provide an integrated review of this study protocol based on both public health and urban planning points of view. The authors of this protocol introduced their proposed prevention program to address the unhealthy urban development issue as a root cause of non-communicable diseases (NCDs). In brief, the authors tried to fill the gap in knowledge and executive steps of inappropriate urban development with this research protocol. Although the project and the provided protocol are well thought and designed, we found many major concerns regarding the proposed plan, which comments are as follows in this letter. Additionally, we read the previous comments of the reviewer and the responses to those comments and tried not to emphasize duplicate issues; however, in some cases, it is inevitable as the issue is major and could not be neglected.

**Authors’ response:** We are grateful to the reviewers for taking the time to feedback on our Protocol with apologies for delay in response. The reviewers’ concerns are understandable and expected, given the wide range of interconnected knowledge domains involved. There is an inherent challenge for journals in assessing research that aims to address complex systemic challenges upstream and brings together many multiple knowledge domains ( Bammer G, 2013).

**Reviewer 2 comment:** Overall, the draft is too complicated. We understand the nature of such work necessitates providing this much information. However, scientific writing always comes alongside simplicity besides the right and essential components of science, and in case of complexity, even some simple points could be missed and make the research less advantageous.

**Authors’ response:** Regarding it being too complicated, we suggest this is because a number of the knowledge domains lie far outside traditional public health and urban planning (e.g. real estate finance, political science, corporate decision-making, law) so it requires an understanding of these other areas to see the implicit linkages within this brief text. Also, the approach is based fundamentally on co-creation, so as the first reviewer observes, it’s not possible to second guess what will emerge. We have been tasked by our funders, the UK Prevention Research Partnership, to develop ‘new approaches to population health research’; in other words, this type of research is still new (Skivington et al, 2021). Where there are specific suggestions for how to simplify, we have taken them on board, but we are unable to respond to generalised concerns.

**Reviewer 2 comment:** Regarding the sections of protocol papers, the format varies based on submitted journals. However, following rational formatting is necessary anyway. Section and subsections of this paper could be divided into three major sections of “Introduction and rationale”, “Protocol”, and “Discussion and conclusion” for a better understanding. It seems that the authors could not follow the coherent presentation of data in the protocol section and the subsections are out of order and this is where the audience of the paper gets bored and confused. Absolutely a revision of the core of this manuscript which is the “Protocol” section could help the reader of this paper.

**Authors’ response:** We contest that presentation is not coherent, and are sorry to hear that you became bored and confused. We do follow the Wellcome Open Access guidelines, which says too that it is ‘flexible’. However, in order to respond to this concern we have moved ‘Research Setting’ to the top, and moved the section on ‘Ethics and Risks’ to after the ‘Analysis Plan’.

**Reviewer 2 comment**: “Introduction and rationale”: this section lacks the necessary information about the burden of NCDs in the real world, especially the investigated country, the UK. In fact, similarly, all parts of the paper lack the proper and essential views of public health experts and this is a major shortcoming of the current draft. The provided material is mainly for urban planning experts and policymakers; however, the goal of this project is to reach optimum health among the population. Therefore, adding some details and the language of public health to this manuscript is necessary in this regard, mainly in the introduction and discussion sections, where the importance of this topic and the ultimate results of this project should be highlighted.

**Authors’ response:** We agree that information on the burden of NCDs is limited, but this is necessary/intentional, and for two main reasons: i) a key requirement is that these protocols are brief so we are restricted in the level of detail of each of the knowledge domains we are uniting (not just information on NCDs); ii) we anticipate that those who read these publications will already be largely familiar with the now relatively well-known population health issues (issues of pollution, mental health, obesity, diabetes are well understood, even by the public in the UK). The material is not provided for urban planning experts alone, but a much wider range of experts. In fact, we are aiming that this is read by those outside of public health. That said, we have added a small amount of additional information on the burden of NCDs in the UK and we have signposted the reader to further information on our public-facing blog, which simplifies the NCD challenge area.

**Reviewer 2 comment**: “Protocol”: as the major section of this protocol, this section may cause significant confusion in readers. The information presented in each part of the work packages (WPs) is too much and does not follow a coherent format. A revision of this section to make it more understandable and only bringing practical points in this protocol paper could help more.

**Authors’ response:** See above.

**Reviewer 2 comment**: One major concern about this protocol is about the period of this project. Based on available information about the current project, it is planned for five years of action starting in 2019, and at this time, about two years have passed. Publication of a protocol of such a huge project, while it reached about half the way, is an alarming concern. In fact, protocols are meant to provide a clear vision of a prospective project, and publishing a complicated protocol like this in the current approved and executed format could be less useful for the scientific communities of both public health and urban planning. However, some amendments in this manuscript by providing the report and results of what the investigators did in these two years and the achievements they accomplished may be helpful to enable the audience to judge the feasibility and efficacy of such a program. For example, the state and results of the designed workshops mentioned in the protocol should be provided in this manuscript. Since a major time has passed, a progress report is necessary.

**Authors’ response:** This protocol was published just over a year into the programme of research, not two: it was published very early in 2021 and the project formally started late in 2019. 2020 was also the year Covid first hit so there have been understandable delays to the programme due to (e.g.) university recruitment freezes. As such, it was still in the very early stages of the 5-year programme when it was published and could not have been published any sooner. The proposal also went through a form of peer review via the application process itself; the protocol therefore is a formalisation of the application form, which was approved by a large advisory group of public health and linked experts. With regards amending the manuscript by providing a report on our first two years of activity, we provide comprehensive annual reports to our funders annually (each around 30 pages in length), which is reviewed each year by that same advisory group and a separate External Advisor Board, made up of leading experts in a wide range of areas both academic and practice based, who we meet with on a quarterly basis. It’s possible we could provide a short summary from these reports – the executive summaries – to add to this Protocol, but it’s not clear, given the complexity, how this information would provide the reviewer with substantive additional information, nor whether the reviewer would expect the same for all future annual reports. It's also not clear why the reviewer specified the state and result of the designed workshops specifically, as the bulk of the data from Phase 1 is derived from the interviews and these are all being written up and published separately. We also have several more detailed design protocols for specific aspects of the evolving research, including the phase 2 intervention design and evaluation, which will be published separately. We will also be providing short and accessible progress reports on our website alongside the range of papers described.

**Reviewer 2 comment**: Similar to the previous review, there is still concern about the two cities chosen for the implementation of policies proposed in this protocol. Authors need to clearly state why they chose the mentioned cities for the specific policies. What made the cities special for running the proposed ideas in this project should be elaborated for readers. This point is very important regarding making the study reproducible in other regions and countries of the world.

**Authors’ response:** We agree that the rationale for the city selection is important and indeed it was a key area that the UKPRP review panel probed us on in the application in 2019, so can repeat our response to them: “ *The size, level of resource, urban health challenges and political denomination of Bristol and Greater Manchester are highly representative of the UK’s other Core Cities - Birmingham, Cardiff, Newcastle, Sheffield, Liverpool, Nottingham, Leeds and Glasgow - the combined population of which is over 20 million (only Glasgow has currently a Lib Dem Leadership; all others are Labour-Led). We are partnering on this project with the Town and Country Planning Association who established the New Communities Group in 2009 with the Department for Communities and Local Government which is made up of 23 ‘ambitious local authorities and development corporations planning and delivering exemplary large-scale new communities* *”*. *We agree it is important that we understand the differences between the various urban authorities and have included representatives from those authorities on our stakeholder groups*.” A final point on this is that we focus on global regions similar to the UK (e.g. OECD nations) as a filter in our search criteria given certain contextual parallels, while also acknowledging the limitations of such broad comparisons.

**Reviewer 2 comment**: “Discussion and conclusion”: as mentioned before, an essential part is missing in this section talking about the potential public health effects of this protocol. A logical estimation of the results and impacts of the implemented policies.

**Authors’ response:** The final paragraph of the discussion and conclusion section addresses this as much as is possible given the research is based fundamentally on co-production: “ *Within a five-year research project, it will not be possible to demonstrate reduction in NCDs…Our goal therefore, is to test demonstrable and immediate changes in attitudes and behaviours amongst the target user groups, as well as changes to policy and practice, within the award period; in particular in decision-making within the systems of governance in Bristol, Manchester (and London in relation to national-level mechanisms linked to city development).*” We appreciate this may sound far less specific than a traditional public health intervention (where intervention areas may already be pre-determined), but the first phase of our programme is focused on mapping and understanding the system, and intervention selection forms the first part of our co-production process, so we are not able to predict up front where exactly we will be intervening, and therefore we can only state in broad terms what kinds of impacts we are expecting (e.g. instrumental, conceptual vs capacity-building). Even the target sectors are unknown: we may focus our interventions into a range of decision-making contexts: e.g. procurement, spatial planning, law, land disposal. We are now in the process of starting that selection process, after which we can start to specific more narrowly the intervention areas, actor constellations and likely impacts.

 _________

  

- Bammer G (2013) Disciplining Interdisciplinarity: Integration and Implementation Sciences for Researching Complex Real-World Problems. ANU Press. Sydney.
- Skivington et al (2021) A new framework for developing and evaluating complex interventions: update of Medical Research Council guidance. Research Methods and Reporting. *BMJ*. Accessed 22/6/22. Available from: https://www.bmj.com/content/bmj/374/bmj.n2061.full.pdf

# Peer review: Reviewer response for version 1

**DOI:** 10.21956/wellcomeopenres.18021.r42640

**Published:** 2021-03-02

**Authors:** Martin McKee

**Affiliations:** 1 London School of Hygiene and Tropical Medicine, London, UK

I find it very difficult to provide a meaningful review of this protocol. First, the project has already been through an extensive process of peer review. Almost inevitably, additional reviewers will have their own preferences about what might be emphasised, added, or subtracted, but given the extensive planning that has already gone into such a complex project, it is difficult to see how this will be particularly helpful to the research team. This is even more so when I read that the research programme started on the 1 October 2019.

 Second, as this particular proposal places a very great emphasis on co-creation with local communities, it is difficult for an external reviewer to second guess what will emerge. There are many questions which one might wish to ask but, realistically, may not be answerable at this stage in the research. I am also conscious of the importance of brevity and I consider it unrealistic to expect the authors to provide the mass of detail that I’m sure they already have. As a consequence, I have limited my comments to those aspects of the proposal that I felt could usefully be clarified, as seen from the perspective of someone reading about the project for the first time.

 My first impression is that there are an awful lot of ideas packed into this protocol, and not always as clearly structured as I felt they might be. Commercial determinants of health feature prominently and, linked to that, power asymmetries. If space permits, it would be helpful to elaborate on this a little further. For example, we know that developers have been very effective in lobbying to influence the planning rules in England. Perhaps something more could be written on this. Linked to this, as we have seen during the pandemic with pressures to reopen office space, the meaning of property in certain discourses has changed. Once, it was about providing shelter, in which people could live or work. Now, for many people, it is an investment vehicle. Again, the consequences of this change, while implicit in some of what is written, could be developed further. For example, they mention how the problems and solutions are to be found in areas such as international finance and investment. This will be very clear to anyone reading the property pages of, for example, some Asian newspapers where the UK property market features prominently as an investment opportunity, something that has contributed to the high level of empty properties in some parts of the country, coinciding with high levels of homelessness. These considerations and others take us to the crucial issue of power. I’m sure that the research team have thought a lot about this, but it would be good to know more of their thinking.

 Given the centrality of power, I wonder if the team have ways of capturing the relevant dynamics, particularly when some aspects of the decision-making are likely to be hidden, either in informal meetings or shielded behind commercial in confidence restrictions? We also know that major capital developments are often an invitation to corruption, favouritism, or to coin a contemporary popular term the “chumocracy”. Has this been considered?

 A related issue is the political dimension. Both Manchester and Bristol are held by Labour, while central government is Conservative. As was seen in the midst of the pandemic in disputes between Manchester and London, this can be problematic. Has this been taken into account? And how?

 The section on valuation was tantalising but brief. I accept that the proposal envisages a substantial body of work to develop thinking on this issue, but again it would be good to have a little more detail of their thinking. Some contemporary thinking is referenced, such as the work of Raworth and Mazzucato, but at present we simply have a list of references to their work without any discussion of what ideas have emerged from their reading of it.

 Public involvement is crucial. However, the public is far from homogeneous and the sorts of interventions that are being considered will bring winners and losers. Put bluntly, policies and housing developments that create more heterogeneous communities, however measured, will be welcomed by some but not by others. How will they address the inevitable tensions that may arise with an advisory board of 6 to 12 members?

 I did feel that the section and dissemination of findings could be elaborated a little more. There is now a wealth of opportunities for communicating the findings of research going far beyond the traditional outlets of peer-reviewed journals. Given the importance of community engagement in this project, it would be interesting to know if the team have come up with any innovative ideas for making this work.

 In summary, this will be a very interesting project and, given the iterative nature of its development, it is unrealistic to expect too much detail at this point. However, I hope that my comments help in pointing to areas where the protocol could be clarified a little more.

Is the study design appropriate for the research question?

Yes

Is the rationale for, and objectives of, the study clearly described?

Yes

Are sufficient details of the methods provided to allow replication by others?

Partly

Are the datasets clearly presented in a useable and accessible format?

Not applicable

Reviewer Expertise:

Public Health.

I confirm that I have read this submission and believe that I have an appropriate level of expertise to confirm that it is of an acceptable scientific standard, however I have significant reservations, as outlined above.

# Response

**Published:** 2021-04-02

**Authors:** Daniel Black

**Affiliations:** University of Bristol, UK

**Reviewer comment:** I find it very difficult to provide a meaningful review of this protocol. First, the project has already been through an extensive process of peer review. Almost inevitably, additional reviewers will have their own preferences about what might be emphasised, added, or subtracted, but given the extensive planning that has already gone into such a complex project, it is difficult to see how this will be particularly helpful to the research team. This is even more so when I read that the research programme started on the 1 October 2019. Second, as this particular proposal places a very great emphasis on co-creation with local communities, it is difficult for an external reviewer to second guess what will emerge. There are many questions which one might wish to ask but, realistically, may not be answerable at this stage in the research. I am also conscious of the importance of brevity and I consider it unrealistic to expect the authors to provide the mass of detail that I’m sure they already have. As a consequence, I have limited my comments to those aspects of the proposal that I felt could usefully be clarified, as seen from the perspective of someone reading about the project for the first time.

**Authors’ response:** We appreciate the time taken by the reviewer to consider our study protocol and very much welcome his comments below. Given the wide-ranging complexity of the subject matter, we have limited ourselves to responding to these reviewer comments here rather than amending the Protocol, which could become unwieldy if we expand on all points touched upon.

**Reviewer comment:** My first impression is that there are an awful lot of ideas packed into this protocol, and not always as clearly structured as I felt they might be.

**Authors’ response:** We agree that the Protocol is packed full, and that there is a good deal sitting behind it. We have sought to describe as simply as possible the complex range of interacting factors influencing these multiple urban systems, but appreciate that it provides only a highly superficial overview encompassing a wide range of areas, and that, as a result, some aspects may appear disparate and disconnected due to the many links that are implicit only to those familiar with the context. We do not have a ready solution to the challenge of structuring the protocol more clearly: the complex nature of the societal challenge is such that, we would argue, it won’t allow for that easily within the limited space available, though we would be pleased to respond to specific areas of change needed. The method and findings papers from the UPSTREAM pilot, which are due for publication at the beginning of April 2021, should provide considerable additional context. A quote from that paper’s abstract, which struggles with the same issue, echoes the points made here: “ *the value of this broad, systems-wide approach is not in the depth of disciplinary investigation, but in its breadth, and the opportunity to draw together multiple strands from multiple systems, thereby balancing complexity and the reduction of that complexity”* (De Pinho, 2018).

**Reviewer comment:** Commercial determinants of health feature prominently and, linked to that, power asymmetries. If space permits, it would be helpful to elaborate on this a little further. For example, we know that developers have been very effective in lobbying to influence the planning rules in England. Perhaps something more could be written on this.

**Authors’ response:** We find that the ‘commercial determinants of health’ framing resonates well with the world of urban development, as it appears to do with unhealthy commodities. The recognition of the dominance of the private sector in UK urban development, and specifically in large-scale property and infrastructure development, is one of the central features of this study. While lobbying and power were not specific themes investigated in the UPSTREAM pilot, commercial factors relating to urban health, and associated aspects of power (and control - e.g. access to land), were revealed across a number of areas: e.g.

- the implications of short-termism on quality of urban environments (estate management, stock market investor interest);
- the challenge of front-loading essential infrastructure investment for long-term health in the face of narrow and short-term viability appraisal;
- the lack of equity stake-holding (which ultimately dictates control) by public agencies and citizens
- justifying public space management budgets within existing models of urban development, planning and management

Power and power asymmetries are central themes within the project. For instance, in Phase One, we are creating our samples from individuals who are deemed to be influential within and knowledgeable about the system. Our focus is thus to talk to the power elite in an effort to understand the system, but also to make these power structures, and thus the asymmetries they create explicit so that they can be understood and challenged.  We are also currently recruiting a PhD to look specifically at understanding the power structures of the system, but also how to best intervene at individual, group, and institutional levels.

 In WP4 (lay public engagement) we are interested explicitly in issues of inequality and ‘citizen voice’, which also overlaps substantially with issues of power.

 It’s perhaps worth flagging that the TRUUD programme is the focus not just on large-scale property development, but also city-region transport, where the role of the private sector is arguably less visible, and yet regardless there are significant economic pressures alongside questions of inequality, which relate to commerce and power (e.g. the challenge of tackling congestion due to the inevitable tensions between winners and losers).

 We also recognise the different and hybrid forms of commercial power exerted by a range of actors in a neo-liberal political economy: e.g. professional architects, planners, designers, marketing, facilitating the circulation and accumulation of capital via built development; city authority collaborations with developers to attract inward investment to cities through the commodification and ‘branding’ of urban space; property, public and private, and pension funds in an investor society.

 In WP4 (lay public engagement) we are interested explicitly in issues of inequality and ‘citizen voice’, which also overlaps substantially with issues of power. In particular we will explore how traditional hierarchies of evidence marginalise the knowledge of local people, which is nevertheless crucial to understanding how the built environment, human agency and health interact. We will exploring creative ways of presenting lay evidence to decision makers as a way of challenging this form of epistemic inequality

**Reviewer comment:** Linked to this, as we have seen during the pandemic with pressures to reopen office space, the meaning of property in certain discourses has changed. Once, it was about providing shelter, in which people could live or work. Now, for many people, it is an investment vehicle. Again, the consequences of this change, while implicit in some of what is written, could be developed further. For example, they mention how the problems and solutions are to be found in areas such as international finance and investment. This will be very clear to anyone reading the property pages of, for example, some Asian newspapers where the UK property market features prominently as an investment opportunity, something that has contributed to the high level of empty properties in some parts of the country, coinciding with high levels of homelessness. These considerations and others take us to the crucial issue of power. I’m sure that the research team have thought a lot about this, but it would be good to know more of their thinking.

**Authors’ response:** With regards the shift from property as shelter to property as investment, this too is a highly complex area with a range of development, investment and delivery issues to bear in mind. The reviewer rightly flags issues caused by recent more rapid changes in global real estate investment - enabled in large part through digitisation of real asset trading (e.g. via Real Estate Investment Trusts (REITs)), the linked internationalisation of investment capital - and the associated flows of global capital into major cities, especially London in the UK, which is having considerable impact on property prices and issues of affordability. The return to public and private investors on property – high-value commercial offices, residential and mixed-use – is extending to niche residential suburban area, e.g. elderly, special needs and social housing. This overseas investment in UK property - as investment vehicles - is driven in large part by the UK's persistent current account deficit. We require overseas investment into the UK and the sale of UK assets to close the gap in our current account caused by deindustrialisation. On the other hand, property investment with a longer-term view (e.g. patient capital) is also seen as critical if we are to enable medium-high density development that can promote walking, cycling and public transport.

 With regards political narratives, property investment is mostly justified in political discourse by housing supply imperatives - i.e. the need to build to meet demand. Political struggles then take place at a local or city level over the affordability of that supply, social segregation in developments, and unmet wider housing needs - rather than questioning the imperative to build more, per se. However, the dominant housing or property discourse is being increasingly challenged by those who argue that affordability problems are caused by the financialisation of housing, not excess demand over supply. The UK's housing supply model is also shaped by a political assumption that the public sector should have a minimal role in delivery of urban infrastructure, and yet the state has nonetheless a major role in supporting the balance sheets of the volume property developers and in maintaining house prices, through policies such as Help to Buy and Stamp Duty holidays.

 A deeper point being made by the reviewer - which is also of real interest to our group and is now part of a wider societal discussion across multiple sectors related to urban development - relates to cultural norms about what is acceptable business practice in hybridized stakeholder assemblages – local authorities, quasi-public sector developer-housing management associations, consultancies, brokers, promoters, landlords, owners, etc. Again, as per the discussion section of the UPSTREAM findings paper, the use of land as a means of generating “unearned income” was decried by none other than Adam Smith and Winston Churchill. The extent to which we can get to grips with all these highly complex areas is to be seen. As above, we are consciously spreading ourselves relatively thinly in order to take a more comprehensive systems view, and in so doing we will be limited in terms of how deeply we can dive in to any given area.

**Reviewer comment:** Given the centrality of power, I wonder if the team have ways of capturing the relevant dynamics, particularly when some aspects of the decision-making are likely to be hidden, either in informal meetings or shielded behind commercial in confidence restrictions? We also know that major capital developments are often an invitation to corruption, favouritism, or to coin a contemporary popular term the “chumocracy”. Has this been considered?

**Author’s response:** This is an important question for a tricky areas that we are very mindful of. Of particular interest is actor-network centrality and the ‘black box’ intermediation spaces where largely ‘off the record’ negotiations take place, e.g. discretionary planning pre-applications, planning agreements for large investment reduced planning risk, CIL, s106 and development viability analysis. Over and above our work on economic valuation, the bulk of our data gathering is focused on qualitative interviews (200+), workshops and industry roundtables (20+) with a wide range of stakeholders. We also have two participant observers working with the relevant councils. We will use the interviews to map actor networks, as far as possible to include those that are hidden within informal settings. But we will also need to use documentary evidence where it exists, including public records (e.g. of Ministerial meetings, registers of interests, donations to parties.) In our experience, using multiple, and different, data sources, tends to mean that at least some of these hidden areas are hinted at, giving us an opportunity to follow up as best as possible. These hidden areas can be challenging to uncover, but it is also the case that some participants, typically in interviews or participant observer work, can be quite candid, giving us glimpses of the hidden world. Combined with an experienced team who already has some knowledge of hidden discussions and negotiations, we hope to be able to dig deeper, making them explicit. In terms of the tools to do this, they include well thought through sampling principles for our starting purposive sample and a snowball sample focused on identifying influential people and structures, detailed field notes, open questions enabling participants to speak freely, creation of safe spaces for communication, and by aggregating and analysing the information across multiple stakeholders. This of course while ensuring that findings made public do not compromise those wishing to be more outspoken. Through this approach, we aim to bring to the surface as many of the barriers and opportunities as possible.

**Reviewer comment:** A related issue is the political dimension. Both Manchester and Bristol are held by Labour, while central government is Conservative. As was seen in the midst of the pandemic in disputes between Manchester and London, this can be problematic. Has this been taken into account? And how?

**Authors’ response:** While writing the research proposal we were aware of the potential political divergence between cities and national government, and across local authorities in large, combined authority areas, (illustrated by CA strategic spatial plan fractures), and it is something we are taking in to account. Our justification for the focus on major urban centres was purely practical. Firstly, they are where most property infrastructure development and transport management takes place. Secondly, we are interested specifically in what cities can do for themselves themselves with devolved powers and budget (but with central government still ‘holding the strings’). Despite the focus on what cities can do for themselves, the role of central government is inevitably critical. Those leading our national level engagement are aware of potential tensions and inevitable obstacles, as they are of the symbiosis between city and state (given they are of the UK’s major economic engines) and the need to work across party lines. Ensuring representation across parties is of course a critical component of the engagement strategy. We will utilise insights from studies of Multi-Level Governance to capture the distribution of power and influence across different tiers of government, and the political tensions to which it can give rise (i.e. when policy priorities diverge, or public spending is cut by central government). Political short-termism in central government, voting system battle lines, government restructuring and centralisation, add layers of complexity to our realm of enquiry.

**Reviewer comment:** The section on valuation was tantalising but brief. I accept that the proposal envisages a substantial body of work to develop thinking on this issue, but again it would be good to have a little more detail of their thinking. Some contemporary thinking is referenced, such as the work of Raworth and Mazzucato, but at present we simply have a list of references to their work without any discussion of what ideas have emerged from their reading of it.

**Authors’ response:** With regards valuation there is another paper forthcoming from the UPSTREAM pilot, in addition to the two on interview method and findings, which is devoted purely to the economic valuation. As above, we appreciate that the information provided in the Protocol is superficial. The approach we are taking has been developing across a number of prior studies, including two commercial feasibility studies (on climate risk valuation) funded by InnovateUK and the Natural Environment Research Council. Legally required social valuation in public sector commissioning is a specific relevant area of enquiry, in the affordable housing sphere. The challenges of quantification and monetisation of qualitative data are very much on our radar. No academic papers were required for the commercial feasibility studies, but they provided us with important lessons in enabling decision-making that places value on socio-environmental (and health) outcomes, which are not easily quantified: e.g.

- Single issue risks must be considered as part of a comprehensive risk mapping (e.g. welfare reform a more pressing risk than climate change)
- Individual beliefs of decision-makers are fundamental (e.g. climate denial)
- Timescales of decision-making are critical (e.g. NHS works to 1-2 year time horizons)
- Quantifiable evidence is highly uneven (e.g. lots on air pollution, limited on overheating)

A final point is that we are aware of the inherent limitations of purely quantifiable economic valuation, and we are hoping in TRUUD to expand our approach beyond quantum, to include more qualitative assessment of value, whether that’s a consideration of political risk, or moral and ethical considerations, for example). There is a rapidly growing industry in the field of social valuation, which this approach will feed in to, and we hope improve.

**Reviewer comment:** Public involvement is crucial. However, the public is far from homogeneous and the sorts of interventions that are being considered will bring winners and losers. Put bluntly, policies and housing developments that create more heterogeneous communities, however measured, will be welcomed by some but not by others. How will they address the inevitable tensions that may arise with an advisory board of 6 to 12 members?

**Authors’ response:** We agree that the public is indeed far from homogenous and the idea of winners and losers is never far from our minds. These tensions will inevitably arise in the advisory board, but even more so within WP4, which is focused on public engagement with the system of decision making. Our advisory board is only one aspect of our public involvement which is designed to ensure that the public have a voice in the governance of TRUUD. In addition to this we will be working with local community organisations in Bristol and Manchester to help us identify and work with underserved communities. The precise selection of which communities to work with will be determined by the emerging focus of our work. Inevitably there will be conflict but our guiding principal would be to act, where possible, to reduce inequality, including inequality in terms of political voice. It is also important to clarify the meaning of representation in these circumstances. In developing detailed plans for our public involvement in WP4, we’ve put forward four types of representation, of which our focus is on the latter two:

1. *statistical* (e.g. quant. research/citizen assemblies);
2. *political* (councillors);
3. *geographical* (e.g. locals, those impacted by specific development proposals)
4. *experiential* (i.e. those affected by a specific aspect of the built environment, such as air pollution)

Thus, we do not envision the advisory board being the only place where concerns, challenges and differences of opinion will come to the attention of the team. The research design, implementation and outputs are being co-produced. It is our hope that we can continue to work with relevant constituents through the process to ensure that our interventions have more lasting and meaningful impact on NCDs and health inequalities. Co-production is not without its challenges, and in WP4 in particular, a great deal of thought has gone into what co-production means, and how it can be practically achieved. We fully anticipate differences of opinion and are aware that there are inevitable winners and losers – see above example on congestion charge - and indeed they are crucial in ensuring any potential conflicts are aired in order for them to be resolved enabling potential interventions to be tested for their efficacy. We’re not yet at a stage in the programme where we can identify the details of the intervention or specific potential changes in policy and practice and so can not foretell either the stakeholders or the possible tensions, but we will seek to ensure, as much as possible within the constraints of the programme, that representation is met and opportunities for discussion fully provided.

 It’s also worth underlining perhaps that the rationale for our approach to public engagement was based in large part on the understanding that very significant amounts of public engagement and involvement already takes place in urban development, and so the core purpose of the public engagement work is to develop a method of disrupting existing means of public engagement, with a focus on inequality and the use of the creative arts.

**Reviewer comment:** I did feel that the section and dissemination of findings could be elaborated a little more. There is now a wealth of opportunities for communicating the findings of research going far beyond the traditional outlets of peer-reviewed journals. Given the importance of community engagement in this project, it would be interesting to know if the team have come up with any innovative ideas for making this work.

**Authors’ response:** We agree that far more needs to be done within academia to better communicate findings, and not just via blogs, conferences and the limited social media work. A key part of our strategy in this area is the nature of the research itself: co-production of the research with a wider range of stakeholders, not just local government partners, but private sector practitioners, third sector membership bodies and the lay public. Beyond that, we are seeking to develop our in house capacity in this area, bearing in mind that research communications tend to be relatively reactive, slow and focused primarily on the academic network. A key focus will be on linking our Theory of Change and emerging impact strategy to the emerging communications, engagement and impact strategies, the objective being to demonstrate what is being done differently to make a positive change.

**Reviewer comment:** In summary, this will be a very interesting project and, given the iterative nature of its development, it is unrealistic to expect too much detail at this point. However, I hope that my comments help in pointing to areas where the protocol could be clarified a little more.

**Authors’ response:** Very helpful indeed, thank you, and likewise, we hope that our answers here are satisfactory. Your time input is greatly appreciated.
